# Long‐Term Efficacy of Phacoemulsification Combined With Trabeculectomy for Advanced Chronic Primary Angle‐Closure Glaucoma With Cataract: A 3‐Year Retrospective Study

**DOI:** 10.1155/joph/3442493

**Published:** 2026-10-14

**Authors:** Chunxin Lai, Baodi Yang, Suwen Zhao, Ling Li, Shuilian Chen, Yongyi Niu, Junhong Fang, Yongjie Qin, Hongyang Zhang

**Affiliations:** ^1^ Department of Ophthalmology the People’s Hospital of Baiyun District Guangzhou China; ^2^ Department of Ophthalmology The Second Affiliated Hospital School of Medicine The Chinese University of Hong Kong, Shenzhen & Longgang District People’s Hospital of Shenzhen Guangdong China; ^3^ The First School of Clinical Medicine Southern Medical University Guangzhou China fimmu.com; ^4^ Department of Ophthalmology Nanfang Hospital Southern Medical University Guangzhou China fimmu.com; ^5^ Department of Ophthalmology Guangdong Eye Institute Guangdong Provincial People’s Hospital Guangzhou China gdghospital.org.cn

**Keywords:** filtering bleb, in vivo confocal microscopy, phacoemulsification, primary angle-closure glaucoma, trabeculectomy

## Abstract

**Purpose:**

To evaluate the long‐term efficacy of phacoemulsification combined with trabeculectomy (Phaco‐Trab) for advanced chronic primary angle‐closure glaucoma (PACG) with cataract.

**Methods:**

Patients with advanced chronic PACG coexisting with cataracts who underwent Phaco‐Trab were enrolled. The morphologic structures of filtering blebs, including characteristics under slit‐lamp examination, anterior segment optical coherence tomography (AS‐OCT), and in vivo confocal microscopy (IVCM), were used to evaluate the filtering function.

**Results:**

A total of 30 eyes from 27 patients were enrolled. The mean preoperative intraocular pressure (IOP) of 19.98 ± 6.48 mmHg significantly decreased to 13.73 ± 2.35 mmHg at year 3 (*p* < 0.0001). A statistically significant decrease in postoperative IOP‐lowering medications was observed, but the number of drugs used increased annually (0.50 ± 0.82 at year 1, 1.00 ± 0.98 at year 2, and 1.47 ± 0.97 at year 3, *p* < 0.0001). Following Phaco‐Trab, a high surgical qualified success rate was maintained over 3 years (93.3% at year 1, 90.0% at year 2, and 80.0% at year 3, *p* = 0.117). However, the complete success rate significantly decreased each year (70.0% at year 1, 36.7% at year 2, and 20.0% at year 3, *p* < 0.0001), along with an increase in the rate of failure filtering blebs, reaching 46.7%, 83.3%, and 90.0% at each time point (*p* < 0.0001). Overall, 46.7% (14/30) of participants developed complications and required postoperative procedures after Phaco‐Trab, which were mainly induced by the trabeculectomy procedure.

**Conclusions:**

Phaco‐Trab can significantly lower IOP and reduce the use of IOP‐lowering medications for advanced chronic PACG with cataracts, but it is associated with a higher rate of complications and a higher failure rate of the filtering bleb.

## 1. Introduction

Primary angle‐closure glaucoma (PACG) is a common cause of blindness, accounting for 50% of glaucoma worldwide and having the highest prevalence in Asians [1]. Eyes with PACG are frequently associated with a shallow anterior chamber, short axial length, a thick lens, and an anterior lens position [2]. Among these factors, a thickened and anteriorly positioned lens mainly contributes to PACG progression by inducing angle closure [3]. Therefore, lens extraction has been commonly performed clinically, and it achieves some curative benefits for PACG patients with coexisting cataracts [4, 5].

However, there is no consensus on the optimal surgical approach for advanced chronic PACG coexisting with cataracts. In practice, patients with chronic PACG are often accompanied by a cloudy lens, making it practical to perform phacoemulsification combined with trabeculectomy (Phaco‐Trab) to treat both diseases in one setting, helping prevent the deterioration of the filtering bleb that could result from subsequent cataract surgery [6]. Previous studies have shown that Phaco‐Trab was more effective than trabeculectomy alone or phacoemulsification alone in controlling intraocular pressure (IOP) in chronic PACG [7–9]. The success of Phaco‐Trab largely depends on the maintenance of the filtering bleb. However, there is limited research on long‐term bleb function. Additionally, with the increasing application of minimally invasive glaucoma surgery (MIGS) in PACG patients, recent findings suggest that phacoemulsification combined with MIGS is also effective and safe for treating advanced chronic PACG within 1 year [10, 11]. Therefore, this retrospective study aims to evaluate the long‐term efficacy of Phaco‐Trab for advanced chronic PACG with cataract and to report on the function of the filtering bleb during 3‐year follow‐up.

## 2. Materials and Methods

This retrospective study aims to investigate the outcomes of Phaco‐Trab for advanced chronic PACG patients at Guangdong General Hospital (GDGH) between November 2013 and July 2021. The study was conducted in accordance with the principles outlined in the Declaration of Helsinki and received approval from the Institutional Review Board at GDGH. Due to the retrospective nature of this study, ethical approval was obtained after the completion of the study period.

### 2.1. Recruitment Criteria

#### 2.1.1. Diagnostic Criteria for PACG [10–12]

1. Angle closure criteria: At least 180 degrees of angle closure, completely obstructing the pigmented part of the trabecular meshwork, whether synechial or appositional, segmented or continuous.
2. IOP criteria: Requiring the use of IOP‐lowering medications or having an IOP above 21 mmHg without IOP‐lowering medications.
3. Glaucomatous criteria: Visual field loss consistent with glaucoma∗ and/or glaucomatous optic disc changes∗∗.

∗Minimal criteria for glaucomatous visual field defect were as follows: (1) glaucoma hemifield test outside normal limits, (2) pattern standard deviation with a *p* value of < 5%, and (3) a cluster of 3 points in the pattern deviation plot in a single hemifield (superior or inferior) with a *p* value of < 5%, one of which must have a *p* value of < 1%. Any one of these criteria, if repeatable, is considered sufficient evidence of a glaucomatous visual field defect. An advanced stage is defined if the patient’s mean deviation (MD) is less than −12.0 dB [13].

∗∗Glaucomatous optic disc changes encompass optic disc atrophy, an increased cup‐to‐disc ratio, nerve fiber layer defects in the peripapillary region, and vascular alterations within the optic disc area, which may manifest as vessel distortion, cupping, or spasm.

*Diagnostic criteria for cataract* were as follows: (1) presence of nuclear sclerosis, cortical cataract, or subcapsular cataract; (2) visual acuity of 20/50 or worse, and impairing activities of daily living.

*Inclusion criteria* were as follows: (1) eyes diagnosed with advanced chronic PACG and concomitant cataract, as defined above; (2) patients who are capable of and willing to provide informed consent for Phaco‐Trab.

*Exclusion criteria* were as follows: (1) patients who declined either cataract extraction or trabeculectomy; (2) previous history of intraocular surgery, except for laser peripheral iridotomy and argon laser peripheral iridoplasty.

As recommended in a previous study, surgical outcomes were defined as follows: (1) complete success: postoperative IOP within the range of 6–18 mm Hg, or a reduction of 20% from baseline, with no loss of light perception, additional ocular hypotensive medication, or reoperation. (2) Qualified success: similar to that of complete success, except for the need for ocular hypotensive medications [14].

### 2.2. Data Collection

Baseline demographic information, including gender, age, and duration of the condition, best‐corrected visual acuity (BCVA) documented as Logarithmic Minimum Angle of Resolution (logMAR), IOP (Goldmann applanation tonometry), and number of glaucoma medications, was collected from all participants. Additionally, a comprehensive set of ocular measurements and assessments were performed, including: range of peripheral anterior synechiae (PAS) using the Scheie system measured under darkroom indentation gonioscopy (O2M, Ocular, USA) by a single professor (HYZ), slit‐lamp anterior segment photography (BX‐900, HAAG‐STREIT, USA), fundus color stereo photography (Canon, Tokyo, Japan), optical biometry measurement (IOL Master 700, Carl Zeiss Meditec, Jena, Germany), ultrasound biomicroscopy (VuMax II, Sonomed, Escalon), visual field MD and pattern standardized deviation (PSD) measured by standard automated perimetry (Humphrey Field Analyzer II 750; 24‐2 Swedish interactive threshold algorithm, Carl Zeiss Meditec, Dublin, CA, USA), anterior segment optical coherence tomography (AS‐OCT) (RTVue‐XR Avanti; Optovue, Fremont, CA, USA, version 2016.2.035), and in vivo confocal microscopy (IVCM) (HRT III Heiderberg Engineering, Germany). Follow‐up data were collected at three time points: 1 year, 2 years, and 3 years after the initial assessment for each participant. These assessments aimed to monitor changes and outcomes over time.

### 2.3. Surgery Procedure

All surgeries were conducted under local anesthesia using 0.5% proparacaine hydrochloride (Alcon) by a single skilled surgeon (HYZ). The surgery commenced with the meticulous creation of a conjunctival flap and a rectangular 5 × 4 mm half‐thickness scleral flap, followed by an antifibrotic procedure (mitomycin [MMC], doses of 0.2–0.4 mg/mL were used for 4–5 min) into the subconjunctival space. Subsequently, sponges saturated with MMC were removed, and the area was thoroughly irrigated with balanced salt solution. Next, a 2‐site procedure of phacoemulsification for cataract removal and the implantation of an acrylic intraocular lens (Akeros Advanced Ai, Bausch & Lomb) were performed. A blade was used to create a peripheral iridectomy; following that, releasable and adjustable 10‐0 nylon sutures were employed to close the scleral flap and conjunctiva. All operated eyes received topical steroids for 4 weeks (dexamethasone eye drops four times a day and dexamethasone 0.15% eye ointment once daily) and topical antibiotic for the initial 2 weeks (levofloxacin 5 mg/mL eye drops four times a day). IOP‐lowering drugs were renewed gradually if an IOP ≥ 15 mmHg (target pressure for advanced glaucoma to avoid progressive visual function loss) at two consecutive follow‐up visits after 3 months postoperatively [15]. Postoperative interventions were conducted if patients were complicated by wound leaking or early filtering bleb fibrosis within 3 postoperative months, including releasable suture removal, digital massage, laser suture lysis, bleb needling with subconjunctival injections of 5‐fluorouracil (5‐FU), and early wound repair [16, 17].

### 2.4. Assessment of Filtering Bleb

Due to the subjective limitations of slit‐lamp classification, recent proposals advocate for the use of AS‐OCT and IVCM to evaluate bleb function at histological and cellular levels [18–20]. Therefore, all filtering blebs in this study were assessed using slit‐lamp anterior segment photography, followed by AS‐OCT and IVCM. Morphologic characteristics of the blebs were recorded according to the Indiana Bleb Appearance Grading Scale (IBACS) to provide a standardized assessment [21]. AS‐OCT was used to investigate the presence of the striping phenomenon, characterized by multiple hypo‐reflective areas within the presumed Tenon’s layer [22]. Microstructures of the filtering bleb, including epithelial microcyst, hyperreflective dots, and stromal connective tissue, were documented using IVCM (HRT III Heidelberg Engineering, Germany) [20, 23, 24]. Five IVCM images were acquired from five regions within the filtering bleb. A single operator (CXL) conducted the IVCM measurements and selected the most representative images from each region, with two other observers (YYN and JHF) responsible for qualitative image measurements. Interobserver agreement analysis was performed between these two observers.

### 2.5. Statistical Analysis

Statistical analysis was conducted using SPSS Sample Power Software (SPSS Inc., Chicago, IL, USA, 27.0). Continuous variables were presented as mean ± standard deviation, and categorical variables were presented as frequencies (percentage). A paired‐sample *t*‐test or Wilcoxon signed‐rank test was used when comparing preoperative and postoperative data. Generalized estimating equations (GEE) were applied to explore the trend of IOP and medication usage during 3‐year follow‐up. Multivariate logistic regression analysis was performed to analyze factors affecting surgical outcomes. Statistical significance was defined when a *p* value (*p*) is less than 0.05 (*p* < 0.05).

## 3. Results

Thirty eyes of 27 participants were included in this study (Table 1). All participants were over 50 years old with an average age of 66.03 ± 7.23 years, and half of them were women (*n* = 14). Patients had a minimum follow‐up time of 3 years. The mean cup‐to‐disc ratio was 0.88 ± 0.12, and all participants’ MDs were less than −12.0 dB, with an average of −24.72 ± 5.79 dB. Patients recruited in this study had relatively short AL (mean of 22.69 ± 0.80 mm) and shallow ACD (mean of 2.41 ± 0.43 mm). As shown in Table 1, all participants exhibited at least half of the clock of PAS preoperatively, and the mean preoperative PAS of 293.00 ± 59.58° significantly reduced to 169.67 ± 92.28° after 3 years of follow‐up (*p* < 0.0001). Significant weak negative correlations were observed with age (*r* = −0.365, *p* = 0.040) and axial length (*r* = −0.365, *p* = 0.048) with postoperative PAS. Preoperative log MAR BCVA of 0.59 ± 0.36 was significantly decreased to 0.34 ± 0.36 postoperatively at year 3 (*p* < 0.0001).

**TABLE 1:** Basic demographics, glaucoma status, and surgical outcomes.

Characteristics	Mean ± SD	Median	Range
Participant (no. of eyes)	27 (30)	—	—
Male/female	13/14	—	—
Right/left	16/14	—	—
Age (years)	66.03 ± 7.23	67.50	50, 77
Baseline C/D	0.88 ± 0.12	0.90	0.50, 1.00
MD (dB)	−24.72 ± 5.79	−24.67	−32.25, −13.04
PSD (dB)	7.86 ± 3.30	9.04	1.59, 12.19
AL (mm)	22.69 ± 0.80	22.68	21.34, 24.57
ACD (mm)	2.41 ± 0.43	2.41	1.62, 3.85
Preoperative PAS (range)	293.00 ± 59.58	300	180, 360
Postoperative PAS (range)	169.67 ± 92.28	150	15, 360
Preoperative BCVA (logMAR)	0.59 ± 0.36	0.52	0.4,2
Postoperative BCVA (logMAR)	0.34 ± 0.36	0.30	0.1,2
Baseline IOP (mmHg)	44.55 ± 9.96	45.65	28.30, 59.00
Preoperative IOP (mmHg)	19.98 ± 6.48	18.50	10.80, 38.00
1 year post IOP (mmHg)	13.21 ± 3.44	13.65	7.00, 20.00
2 years post IOP (mmHg)	13.68 ± 2.11	13.80	10.70, 17.30
3 years post IOP (mmHg)	13.73 ± 2.35	13.85	9.30, 18.30
Preoperative IOP‐lowering drugs (no.)	3.17 ± 0.79	3	1, 4
1 year post IOP‐lowering drugs (no.)	0.50 ± 0.82	0	0, 2
2 years post IOP‐lowering drugs (no.)	1.00 ± 0.98	1	0, 3
3 years post IOP‐lowering drugs (no.)	1.47 ± 0.97	2	0, 3

*Note:* C/D: ratio of cup to disc; AL, axial length, measured by IOL Master 700; ACD, anterior chamber depth, measured by IOL Master 700; Postoperative PAS and BCVA demonstrated records at year 3; IOP: intraocular pressure, data measured by Goldmann applanation tonometry.

Abbreviations: BCVA: best corrected visual acuity; MD, mean deviation; PAS, peripheral anterior synechiae; PSD, pattern standard deviation.

### 3.1. IOP and Medication Reduction

Mean baseline IOP without using IOP‐lowering medications was 44.55 ± 9.96 mmHg, which was reduced to 19.98 ± 6.48 mmHg 1 day before surgery, with a mean number of IOP‐lowering drugs of 3.17 ± 0.79. Following Phaco‐Trab, the mean IOP decreased to 13.21 ± 3.44 mmHg at year 1, 13.68 ± 2.11 mmHg at year 2, and 13.73 ± 2.35 mmHg at year 3, demonstrating a statistically significant IOP reduction in the long term (*p* < 0.0001). No significant difference was found in IOP changes between years 1, 2, and 3 (*p* = 0.425).

The surgical efficacy lasted for 3 years, achieving an absolute IOP reduction of 30.84 ± 9.83 mmHg from baseline. Statistically significant reductions in IOP‐lowering medications were observed at each time point postoperatively (*p* < 0.0001). As shown in Table 2, the proportion of medication‐free eyes increased to 70.0% at year 1 but decreased to 36.7% at year 2% and 20.0% at year 3 (*p* < 0.0001). Additionally, the mean number of IOP‐lowering medications used increased each year significantly (0.50 ± 0.82 at year 1, 1.00 ± 0.98 at year 2, and 1.47 ± 0.97 at year 3, *p* < 0.0001, Table 1).

**TABLE 2:** Crosstab for numbers of IOP‐lowering drugs at each time point.

Time points	IOP‐lowering drugs, *n* (%)					Sum
	0	1	2	3	4	
Preoperative	0 (0)	1 (3.3)	4 (13.3)	14 (46.7)	11 (36.7)	30 (100)
Postsurgery at year 1	21 (70.0)	3 (10.0)	6 (20.0)	0 (0)	0 (0)	30 (100)
Postsurgery at year 2	11 (36.7)	11 (36.7)	5 (16.6)	3 (10.0)	0 (0)	30 (100)
Postsurgery at year 3	6 (20.0)	8 (26.7)	12 (40.0)	4 (13.3)	0 (0)	30 (100)

### 3.2. Bleb Morphology and Function [23, 24]

As depicted in Figure 1, a functional filtering bleb has a cystic, diffuse appearance, exhibiting a striping phenomenon under AS‐OCT, with multiple microcysts and few hyper‐reflective dots in the epithelial layer and lossy connective tissue under IVCM (Figure 1a). However, a failed filtering bleb exhibits a fat, congestive gross appearance, displaying few microcysts and thousands of hyper‐reflective dots in the epithelial layer, together with dense stroma under IVCM (Figure 1b). Interobserver agreement for bleb function grading was high, and the double‐blind repeated grading yielded a Cohen’s kappa coefficient of 0.918 (*p* < 0.0001). In accordance with the above criteria, all the filtering blebs were classified at each time point, with 16 functional blebs (53.3%) and 14 (46.7%) failed at year 1, 5 functional blebs (16.7%) and 25 failed (83.3%) at year 2, and 3 functional blebs (10.0%) and 27 failed (90.0%) at year 3 (*p* < 0.0001).

**FIGURE 1:**
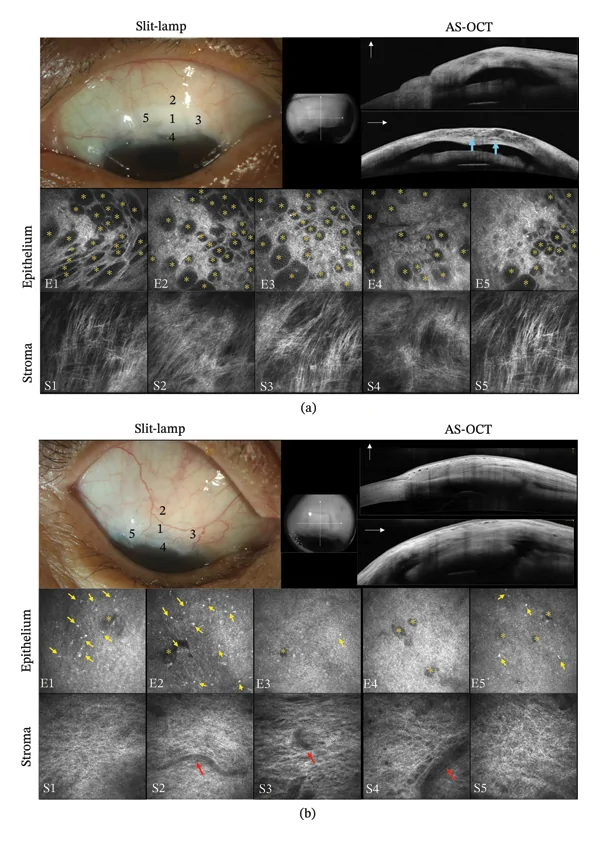
Functioning (a) and nonfunctioning (b) filtering blebs after Phaco‐Trab. Filtering blebs were first evaluated under a slit‐lamp. Then AS‐OCT was taken across the line (white arrow) to find the striping phenomenon, demonstrating multiple parallel hyporeflective layers inside the presumed Tenon’s layer (blue arrow in 1(a)). All participants were scanned from sites 1 to 5 (shown in the photograph) using IVCM. For each scanning site, epithelium and stroma were both analyzed. Microcysts area (yellow star) and hyper‐reflective dots (yellow arrow) were found in the epithelium (E1–E5). Corrugated blood vessels (red arrow) were found in connective tissue (S2–S4 in 1(b)).

### 3.3. Surgical Success Rate

Following Phaco‐Trab, a high surgical qualified success rate was maintained over 3 years (93.3% at year 1, 90.0% at year 2, and 80.0% at year 3, *p* = 0.117), while the complete success rate significantly decreased each year (Figure 2). The percentage of participants maintaining target IOP without medications dropped significantly to 70.0%, 36.7%, and 20.0% at each time point (*p* < 0.0001). Along with this, the failure rate of filtering blebs increased to 46.7%, 83.3%, and 90.0% over the same period (*p* < 0.0001). Additionally, the use of IOP‐lowering medications progressively increased each year (0.50 ± 0.82 at year 1, 1.00 ± 0.98 at year 2, and 1.47 ± 0.97 at year 3, *p* < 0.0001), indicating that the function of filtering blebs gradually diminished or disappeared after Phaco‐Trab.

**FIGURE 2:**
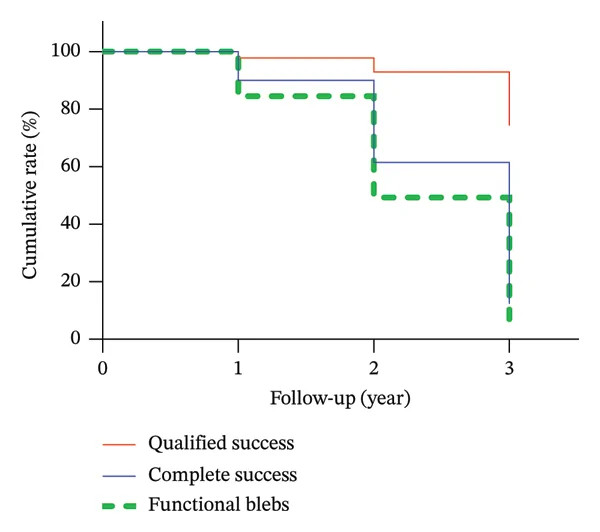
Plot showing the cumulative probability of success and functional blebs up to 3 years postoperatively. Complete success: postoperative IOP within the range of 6–18 mm Hg, or a reduction of 20% from baseline, with no loss of light perception, additional ocular hypotensive medication, or reoperation. Qualified success: similar to that of complete success, except for the need for ocular hypotensive medications.

To identify the factors influencing surgical outcomes for Phaco‐Trab, a multivariable GEE was conducted to explore the relationship between filtering bleb function, postoperative PAS, and surgical success (Table 3). Prolonged duration of follow‐up was a significant risk factor for failure of surgical success. For complete success, the odds of failure increased significantly at year 2 (OR = 2.508, 95% CI = 1.053–5.975, *p* = 0.003) and year 3 (OR = 5.625, 95% CI = 1.818–17.401, *p* = 0.038). For qualified success, the odds of non‐qualified success increased significantly at year 2 (OR = 7.894, 95% CI = 1.871–33.314, *p* = 0.005), but not significant at year 3 (OR = 2.856, 95% CI = 0.874–9.335, *p* = 0.082). Adjusted follow‐up time and functional blebs were associated with a slightly significant higher rate of complete success (OR = 0.143, 95% CI = 0.02–1.023, *p* = 0.053) but not associated with qualified success (OR = 3.705, 95% CI = 0.344–39.925, *p* = 0.280). Besides, postoperative PAS had no significant effect on either surgical success.

**TABLE 3:** Affected factors analysis for surgical outcomes.

Parameters	Complete success		Qualified success	
	OR (95% CI)	*p* Value	OR (95% CI)	*p* Value
Follow‐up times				
Post 1 year	Reference		Reference	
Post 2 years	2.508 (1.053–5.975)	0.003^∗^	7.894 (1.871–33.314)	0.005^∗^
Post 3 years	5.625 (1.818–17.401)	0.038^∗^	2.856 (0.874–9.335)	0.082
Filtering bleb status				
Failed	Reference		Reference	
Functional	0.143 (0.02–1.023)	0.053	3.705 (0.344–39.925)	0.280
Postoperative PAS				
< 180°	Reference		Reference	
≥ 180°	2.655 (0.628–11.232)	0.184	3.774 (0.505–28.204)	0.196

*Note:* Analyzed by multivariate generalized estimating equations (GEEs).

Abbreviation: PAS, peripheral anterior synechiae.

^∗^Statistically significant.

### 3.4. Complications

After Phaco‐Trab, 14 out of 30 eyes (46.7%) developed early complications such as encapsulated bleb or bleb fibrosis within 3 months postoperatively. Additionally, 7 out of 30 eyes (23.3%) experienced fibrin exudation and hypotony, and 6 (20.0%) developed a shallow anterior chamber. And 3 out of 30 eyes (10.0%) developed wound leak and choroidal detachment. Hyphema and cystoid macular edema occurred in 1 out of 30 eyes (3.3%), as shown in Table 4. Moreover, there was a significant burden of postoperative interventions following Phaco‐Trab. Of 30 eyes, 14 (46.7%) underwent releasable suture removal and digital massage, and 3 (10.0%) needed suture lysis. One eye (3.3%) required needling with 5‐FU, and 1 eye (3.3%) required wound repair. Notably, most of the complications mentioned were primarily associated with the trabeculectomy procedure.

**TABLE 4:** Complications and postoperative procedures for Phaco‐Trab.

Complications	*n* (%)^b^
Encapsulated bleb or bleb fibrosis^a^	14 (46.7)
Fibrin exudation	7 (23.3)
Hypotony	7 (23.3)
Shallow anterior chamber	6 (20.0)
Wound leak	3 (10.0)
Choroidal detachment	3 (10.0)
Hyphema	1 (3.3)
Cystoid macular edema	1 (3.3)

**Postoperative procedures** ^a^	***n* (%)**

Releasable suture removal	14 (46.7)
Digital massage	14 (46.7)
Suture lysis procedure	3 (10.0)
Needling with 5‐fluorouracil	1 (3.3)
Repair of wound	1 (3.3)

^a^Complication of encapsulated bleb or bleb fibrosis and postoperative procedures were determined in the first 3 months after Phaco‐Trab.

^b^ *n* (%) indicates the number of complications and procedures. Note that some patients had more than one complication and postoperative procedure.

## 4. Discussion

Angle‐closure glaucoma remains a leading cause of blindness in China [25]. The first‐line treatment of PACG is surgery, and the preferred option for advanced chronic PACG is trabeculectomy. As the demographic predisposed to chronic glaucoma frequently overlaps with individuals susceptible to cataracts, combined phacoemulsification with trabeculectomy has been a common approach for decades [26]. A previous meta‐analysis comprising 14 randomized controlled trials for PACG treatment showed that Phaco‐Trab exhibited favorable outcomes in controlling IOP and reducing antiglaucoma medications used at 1 year after surgery [5]. Research on long‐term surgical outcomes is limited. A 5‐year randomized controlled trial comparing phacoemulsification alone to Phaco‐Trab for PACG with cataract found that Phaco‐Trab was more effective in reducing IOP and glaucoma drugs used but was associated with more complications than phacoemulsification alone [9]. However, this study included patients with early and middle stages of PACG without specifying the severity stage and did not address long‐term function of filtering blebs [9]. Our study confirmed the efficacy of Phaco‐Trab in managing advanced chronic PACG. We found a significant reduction in IOP and glaucoma medication use, consistent with earlier studies [5, 9, 27]. Our results indicated a similar 1‐year cumulative qualified success rate with previous studies for Phaco‐Trab (93.2% vs. 93.3% in our study), but the cumulative complete success rate was lower than previous studies (89.8% vs. 70.0% in our study) [10]. The discrepancy may be explained by our stricter criteria for postoperative IOP control, which necessitated the use of IOP‐lowering medications if IOP was ≥ 15 mmHg at two consecutive follow‐up visits after 3 months postoperatively. The rate of complete success dropped significantly to 36.7% at year 2 and 20.0% at year 3 (*p* < 0.0001). Concurrently, the use of IOP‐lowering medications increased usage of annually (0.50 ± 0.82 at year 1, 1.00 ± 0.98 at year 2 and 1.47 ± 0.97 at year 3, *p* < 0.0001), and the proportion of failed filtering blebs rose dramatically to 46.7%, 83.3% and 90.0% by years 1, 2, and 3, respectively (*p* < 0.0001), indicating that function of filtering blebs diminished or disappeared over time, challenging the long‐term effectiveness of Phaco‐Trab for advanced chronic PACG.

It is generally acknowledged that a successful filtering surgery mainly depends on the function of the filtering bleb. Our study reported that the rates of functional filtering bleb significantly decreased during the follow‐up period and were almost eliminated at the last visit (53.3% (16/30) at year 1, 16.7% (5/30) at year 2, and 10.0% (3/30) at year 3) based on observations of the morphology using IVCM. The functional filtering bleb group maintained lower mean IOP and fewer IOP‐lowering drugs than the failed group (Supporting Table 1). However, our study found no significant difference in the mean IOP, IOP‐lowering drugs, and surgical success rates between the functional and failed filtering bleb groups (Table 3, Supporting Table 1), which might be attributed to the limited sample size of our study.

With a significant reduction in PAS observed postoperatively (preoperative 293.00 ± 59.58° vs. postoperative 169.67 ± 92.28° at year 3, *p* < 0.0001), the internal drainage pathway may also play an important role in controlling IOP after Phaco‐Trab. It has been proposed that the reduction in PAS might enhance internal trabecular drainage, potentially contributing to IOP control by replacing the conjunctival filtration pathway. However, despite both the cumulative qualified success rate and complete success rate being higher in the PAS < 180‐degree group, no significant surgical success rate was found between the two stages of postoperative PAS (*p* = 0.196 for qualified success and *p* = 0.184 for complete success). A weak negative correlation was noted between age, axial length, and postoperative PAS (*r* = −0.365, *p* = 0.040 for age; *r* = −0.365, *p* = 0.048 for axial length), indicating that younger patients and those with a shorter axial length are at a higher risk of developing postoperative PAS.

Recent research indicates that MIGS, including combined phacoemulsification, goniosynechialysis, and goniotomy, effectively reduced IOP in advanced PACG patients with significantly fewer complications [10, 11]. A recent multicenter randomized trial has proved that phacogoniotomy was non‐inferior to Phaco‐Trab in terms of IOP reduction for patients with advanced PACG and cataracts over 1 year [10]. In line with our findings, filtering blebs effectively controlled IOP only during the first year in advanced chronic PACG with cataracts. Furthermore, in our study, about 46.7% (14/30) of participants developed complications and required postoperative procedures following Phaco‐Trab, mainly due to the trabeculectomy procedure. Various previous studies have revealed similar rates of complications and postoperative interventions in the Phaco‐Trab group, but significantly higher rates compared to phacoemulsification alone [28, 29]. Therefore, the necessity of Phaco‐Trab for advanced chronic PACG patients should be further assessed.

The primary limitations of this study include its retrospective design, small sample size (*n* = 30 eyes), and single‐center data. These factors inherently limit the generalizability of our findings, especially the result of non‐significant difference in comparing the surgical success rate between the functional and failed bleb groups. Additionally, the non‐comparable nature of this study may limit its statistical power to comprehensively explore mechanisms of IOP control in PACG after Phaco‐Trab. Recently, research on MIGS has increased significantly, demonstrating the safety and effectiveness of phacoemulsification combined with MIGS in PACG cases. Therefore, the decision on whether patients with concomitant advanced chronic PACG and cataracts should undergo Phaco‐Trab remains a subject of debate.

## 5. Conclusion

In conclusion, Phaco‐Trab is an effective surgical approach for managing advanced chronic PACG with cataracts, offering significant IOP reduction and medication‐sparing effects over 3 years. However, the potential for complications and the eventual decline in bleb function must be carefully considered in the decision‐making process.

## Author Contributions

All authors meet the criteria for authorship. Hongyang Zhang and Yongjie Qin conceptualized and designed the study and had full access to all the data. Chunxin Lai and Baodi Yang enrolled the participants, processed the data, and drafted the manuscript. Suwen Zhao, Ling Li, and Shuilian Chen were responsible for collecting preoperative and postoperative data. Chunxin Lai performed slit‐lamp photography, anterior segment optical coherence tomography, and in vivo confocal microscopy for all participants. Yongyi Niu and Junhong Fang independently classified the functions of the filtering blebs and blinded to study data. Yongjie Qin coordinated the study and revised the initial manuscript draft. Hongyang Zhang performed all surgeries and reviewed and edited the final manuscript.

## Funding

This work was supported by the Science and Technology Project in Baiyun District, Guangzhou (Grant No. 2025‐YL‐005), President Foundation of the People’s Hospital of Baiyun District Guangzhou (Grant No. BYYZ25009), the Clinical Research Fund of Nanfang Hospital, Southern Medical University (Grant No. 2025CR009), the Clinical Research Fund of Nanfang Hospital, Southern Medical University (Grant No. 2025CR009), National Natural Science Foundation of China (Grant No. 82171036), the Natural Science Foundation of Guangdong Province, China (Grant No. 2023A1515011735), and Science and Technology Innovation Special Fund of Longgang District of Shenzhen, China (Grant No. LGKCYLWS2022).

## Disclosure

All authors have read and approved the final version of the manuscript.

## Ethics Statement

This study was performed in accordance with the tenets of the Declaration of Helsinki. It had been approved by the ethics committee of Guangdong Provincial People’s Hospital (NO: XJS2023‐062‐02).

## Consent

All the written informed consent was obtained from the participants, including for the publication of the identifiable images in Figure 1.

## Conflicts of Interest

The authors declare no conflicts of interest.

## Supporting Information

Additional supporting information can be found online in the Supporting Information section.

## Supporting information


**Supporting Information** Supporting Table 1. IOP and IOP‐lowering drugs used in functional vs. failed bleb groups. The functional filtering bleb group maintained lower mean IOP and lesser IOP‐lowering drugs than the failed group in their years but without significant difference.

## Data Availability

The data that support the findings of this study are available from the corresponding author upon reasonable request.
