# Supplementary material for: Long‐Term Efficacy of Phacoemulsification Combined With Trabeculectomy for Advanced Chronic Primary Angle‐Closure Glaucoma With Cataract: A 3‐Year Retrospective Study
Source: J Ophthalmol. 2026 Oct 14;2026:3442493. doi: 10.1155/joph/3442493 (PMC13669659; doi:10.1155/joph/3442493)
Supplement: Supplementary file 1 [file JOPH-2026-3442493-s001.docx]

**Supplementary Table 1. IOP and IOP-lowering drugs used in functional vs. failed bleb groups.**

| Times/year | Blebs function | IOP/mmHg | P value | IOP-lowering drugs | P value |
| --- | --- | --- | --- | --- | --- |
| 1 | Functional | 12.81±3.94 | 0.423 | 0.31±0.70 | 0.275 |
|  | Failed | 13.67±2.85 |  | 0.71±0.91 |  |
| 2 | Functional | 13.60±2.67 | 0.829 | 0.60±1.34 | 0.188 |
|  | Failed | 13.69±2.05 |  | 1.08±0.91 |  |
| 3 | Functional | 13.43±3.61 | 0.897 | 1.00±1.73 | 0.467 |
|  | Failed | 13.77±2.27 |  | 1.52±0.89 |  |

Anaylzed by Mann-Whitney U test.
